# Metabolic Signature of Pluripotent Stem Cells

**DOI:** 10.22074/cellj.2018.5514

**Published:** 2018-05-28

**Authors:** Sara Taleahmad, Seyedeh Nafiseh Hassani, Ghasem Hosseini Salekdeh, Hossein Baharvand

**Affiliations:** 1 Department of Molecular Systems Biology, Cell Science Research Center, Royan Institute for Stem Cell Biology and Technology, ACECR, Tehran, Iran; 2 Department of Stem Cells and Developmental Biology, Cell Science Research Center, Royan Institute for Stem Cell Biology and Technology, ACECR, Tehran, Iran; 3 Department of Systems Biology, Agricultural Biotechnology Research Institute of Iran, Karaj, Iran; 4 Department of Developmental Biology, University of Science and Culture, ACECR, Tehran, Iran

**Keywords:** Cell Cycle, Glycolysis, Metabolism Process, Mouse Embryonic Stem Cells

## Abstract

**Objective:**

Pluripotent stem cells (PSCs), with the capacity to self-renew and differentiate into all other cell types, are of benefit in regenerative medicine applications. Tightly controlled gene expression networks and epigenetic factors regulate these properties. In this study, we aim to evaluate the metabolic signature of pluripotency under 2i and R2i culture conditions versus serum condition.

**Materials and Methods:**

In this experimental study, we investigated bioinformatics analysis of the shotgun proteomics data for cells grown under 2i, R2i, and serum culture conditions. The findings were validated by cell cycle analysis and gene expressions of the cells with flow cytometry and quantitative reverse transcription-polymerase chain reaction (qRT-PCR), respectively.

**Results:**

Expressions of 163 proteins increased in 2i-grown cells and 181 proteins increased in R2i-grown cells versus serum, which were mostly involved in glycolysis signaling pathway, oxidation-reduction, metabolic processes, amino acid and lipid metabolism. Flow cytometry analysis showed significant accumulation of cells in S phase for 2i (70%) and R2i (61%) grown cells.

**Conclusion:**

This study showed that under 2i and R2i conditions, glycolysis was highlighted for energy production and used to maintain high levels of glycolytic intermediates to support cell proliferation. Cells grown under 2i and R2i conditions showed rapid cell cycling in comparison with the cells grown under serum conditions.

## Introduction

Embryonic stem cells (ESCs), have the potential to differentiate into all types of cells along with the capability for self-renewal. According to these properties, ESCs are used in both developmental studies and regenerative medicine. Hence, it is important to understand the mechanism that controls pluripotency maintenance and self-renewal of ESCs. Although the pluripotency network genes and growth factors are important in the determination of a stem cell fate, various metabolic pathways regulates the self-renewal and pluripotency maintenance of cells by changes in energy metabolism (1, 2). 

A recent study documented the rapid and dynamic changes in substrate utilization during early embryogenesis. The results have shown an important role for metabolism in regulating stem cell biology (3). ESCs require rapid cell duplication, therefore a balance must be struck between energetic and biosynthetic demands (4). Changes in cellular metabolism can affect the activity of epigenetic-modifying enzymes (5). In addition, the regulation of energy metabolism appears intertwined with the genetic and epigenetic mechanisms that control stem cell fate through pathways that require further elucidation. Metabolic pathways generate ATP and produce glycolytic intermediates essential for anabolic reactions during cell division and release of metabolites used in enzymatic reactions, which include those involved in mediating epigenetic modifications essential for stem cells function (6). 

Two broad classes of pluripotent stem cells (PSCs) have been isolated from embryonic sources: naïve or ground-state and a primed that have specific characteristics. Mouse ESCs (mESCs) can be preserved in their naïve state when cultured in that medium contains bone morphogenetic protein 4 (BMP4) and leukemia inhibitory factor (LIF) (7), or in the ground state by inhibitors of fibroblast growth factor 4 (FGF4) and glycogen synthase kinase 3 (GSK3) known as 2i (8), and inhibitors of FGF4 and transforming growth factor beta (TGFß) known as R2i (9). Under 2i and R2i conditions glycolysis is more highlighted for energy production, in PSCs, glycolysis is functionally important for maintenance of the pluripotent state. The intermediate products of glycolysis are necessary for stem cell proliferation. 3-phosphoglycerate can be used to make glycine and serine, which are needed in amino acid, lipid, and nucleotide biosynthesis (10). Upon differentiation, ESCs down-regulate glycolysis and oxidize most of the glycolysis-derived pyruvate in mitochondria via oxidative phosphorylation (OXPHOS) (11). In this study, we compare mESCs cultured under 2i and R2i conditions with cells grown under serum condition, and describe the correlation between mESCs metabolism and their maintenance in cell culture conditions.

## Materials and Methods

### Culture of mouse embryonic stem cell

All materials were purchased from Sigma unless otherwise noted. In this experimental study, we cultured three biological repeats of the mESCs line Royan B20 (Royan Institute, Iran) on 0.1% gelatin-coated plates in 2i/LIF, R2i/LIF (serum-free N2B27 medium) and serum/ LIF medium. The 2i treatment included MEK and GSK3 inhibitors PD0325901 (1 µM, Stemgent, USA) and CHIR99021 (3 µM, Stemgent, USA) (8). The R2i culture contained 1 µM PD0329501 and 10 µM SB431542 which inhibited the TGFß signaling pathway (9). N2B27/LIF medium contained a 1:1 ratio of neurobasal (Invitrogen, USA) and DMEM/F12 (Invitrogen, USA), 1% N2 supplement (Invitrogen, USA), 1% B27 supplement (Invitrogen, USA), 2 mM L-glutamine (Invitrogen, USA), 1% nonessential amino acids (Invitrogen, USA), 0.1 mM ß-mercaptoethanol, 100 mg/ml streptomycin (Invitrogen, USA), 100 U/ml penicillin (Gibco, USA), and 5 mg/mL bovine serum albumin. Serum medium consisted of knockout Dulbecco’s modified Eagle’s medium (KoDMEM, Invitrogen, USA), 15% fetal bovine serum (FBS, HyClone, Germany), 2 mM L-glutamine (Gibco, USA), 1% nonessential amino acids, 100 mg/ml streptomycin (Gibco, USA), 100 U/ml penicillin, and 0.1 mM ß-mercaptoethanol. 

### Immunofluorescence staining and alkaline phosphatase detection

Immunofluorescence (IF) was performed after fixation of the cultured cells in 4% paraformaldehyde for 20 minutes followed by permeabilization with 0.2% Triton X-100 (Merk, USA) for 30 minutes. The cells were subsequently blocked in phosphate- buffered saline (PBS) supplemented with 10% secondary antibodies host serum for 1 hour. The blocked cells were incubated overnight at 4°C with mouse anti-Oct4 (Santa Cruz, USA, sc5279), mouse anti-SSEA-1 (R&D, MAB2155) and goat anti-Nanog (Santa Cruz, USA, sc30329). The cells were washed three times with PBS and subsequently incubated with the following secondary antibodies goat anti-mouse IgG-FITC (Santa Cruz, USA, sc2010), Alexa Fluor 568 goat anti-mouse (Invitrogen, USA, A21043), and Alexa Fluor 568 donkey anti-goat (Invitrogen, USA, A11057). The cells were stained with 1 µg/ ml DAPI for 10 minutes in the dark and after three PBS washes, we used an Olympus fluorescent microscope (Olympus, Japan) to visualize the cells. Alkaline phosphatase (ALP) staining was performed according to the manufacturer’s instructions using an Alkaline Phosphatase Detection Kit. 

### Protein extraction, identification, and quantitative proteomic analysis

Total protein of 5×10^6^ cells extracted by Qiagen lysis buffer (Qiagen, Germany) according to the kit’s manual. The concentration of the extracted protein was determined by the Bradford Assay kit (BioRad, Hercules, CA, USA). Samples were then separated by SDS-PAGE on a 12% (W/V) separating gel. Sample preparation for mass spectrometry, protein identification and quantitative proteomic analysis were performed as previously described (12).

### Functional annotation

Functional annotations were obtained as gene ontology (GO) annotations from PANTHER™ (version 12.0) and the Database for Annotations, Visualization, and Integrated Discover (DAVID, version 6.7). 

The differentially expressed proteins include up-, and down-regulated proteins categorized based on the biological process (BP) using the DAVID database. Down-regulated proteins between 2i/R2i conditions versus serum reported in our previous study and in this study we focused on the up-regulated proteins between 2i/R2i conditions versus serum. The enriched pathways were determined by using the Kyoto Encyclopedia of Genes and Genomes (KEGG) database. 

### Quantitative reverse transcription polymerase chain reaction analysis 

We extracted three replicates of the total RNA from the 2i-, R2i-, and serum-grown cells using the RNeasy Plus Mini Kit (Qiagen, Germany) according to the manufacturer’s instructions. cDNA was generated with a High Capacity cDNA Reverse Transcription Kit (Life Science, UK) according to the manufacturer’s instructions.

Transcript level of *c-Myc* (accession number: 010849.4, amplicon size: 175): F: 5´GCCTACATCCTGTCCATTCA3´
  R: 5´AACCGTTCTCCTTACTCTCA3´ and
Hif1a (accession number: 001313920.1, amplicon size: 73): F: 5´ATAATGTTCCAATTCCTACTGCTTG3´
  R: 5´CAGAATGCTCAGAGAAAGCGAAA3´

were determined using the SYBR Green master mix and 7900HT Sequence Detection System (Life Science, UK). 

Data were normalized to the *GAPDG* (accession number: 001289726.1, amplicon size: 113): 

F: 5´CAAGGAGTAAGAAACCCTG3´

R: 5´TCTGGGATGGAAATTGTGAG3´

housekeeping gene and relative quantification of gene expressions were calculated with the △△Ct method.

### Cell cycle assay

The cell cycle distribution was analyzed by flow cytometry. We harvested 2×10^5^ 2i, R2i and serum-grown cells. These cells were washed twice with cold PBS (calcium and magnesium free) and fixed with 1ml of 70% cold ethanol for 2 hours at 4°C. After fixation, the cells were washed twice with PBS (calcium and magnesiumfree), and re-suspended in staining solution [50 µg/ ml propidium iodide (PI), 100 µg/ml RNase A in PBS(calcium and magnesium free)] for 10 minutes at 37°C. Prior to analysis, the cells were incubated with 200 µl of PI(50 µg/ml) for 5 minutes at 37°C. Cell cycle analysis wasperformed on a BD FACS-Calibur flow cytometer and the Cell Quest program (Becton-Dickinson, San Jose, CA).

### Statistical analysis 

Statistical analysis was performed using one-way analysis of variance (ANOVA) and the student’s t test with Fisher’s LSD post hoc tests. P<0.05 was considered to be statistically significant.

## Results

### Morphology and characterization of mouse embryonic stem cells

**Fig.1:**
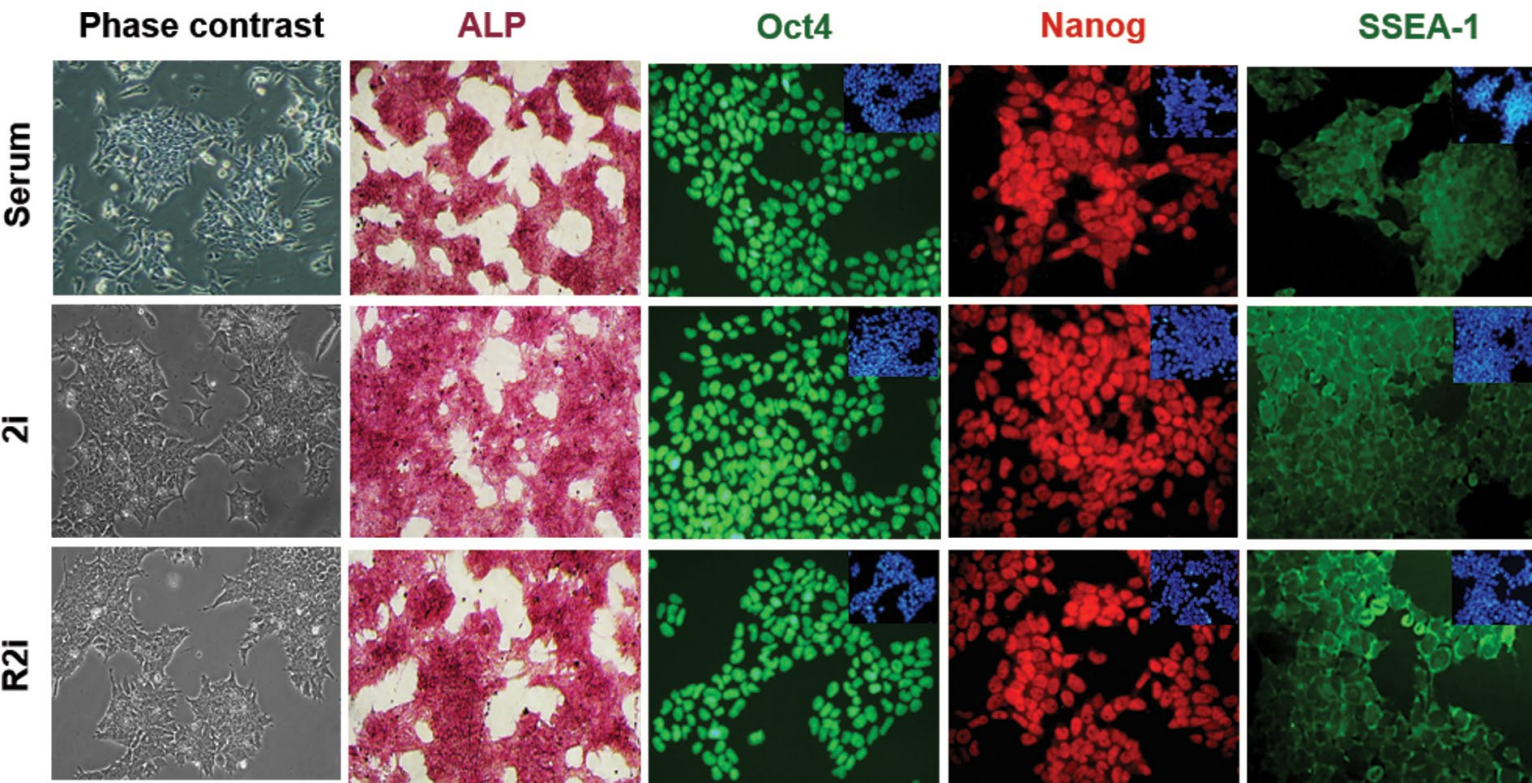
Characteristics of mouse embryonic stem cells (mESCs) cultivated in 2i, R2i, and serum. Alkaline phosphatase (ALP) staining (scale bar: 100 µm) and immunofluorescence (IF) labeling for Oct4, SSEA-1, and Nanog counterstained for DAPI are shown (scale bar: 50 µm).

The mESCs propagated on 2i, R2i and serum medium grew as dome-shaped colonies with typical ESC morphology. These cells also retained expression of key pluripotency markers that included Oct4, Nanog and SSEA-1 (Fig .1). 

### Up-regulated metabolic pathway under 2i and R2i culture conditions 

**Fig.2:**
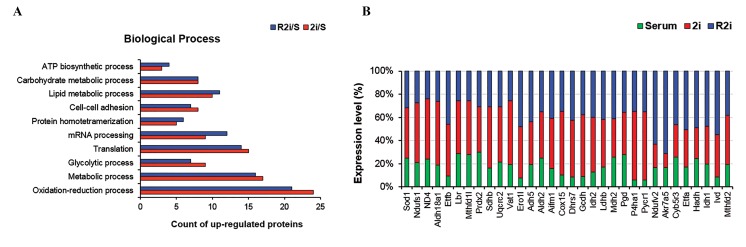
Biological process of up-regulated proteins in 2i- and R2i-grown cells. A. Gene ontology (GO) in the term of the biological process (BP) of up- regulated proteins in 2i- and R2i-grown cells versus serum and B. Protein expressions in 2i, R2i, and serum in terms of the oxidation-reduction process.

We used the shotgun proteomics analysis from our previous study (13) to show 163 proteins in the 2i culture and 181 proteins in the R2i culture significantly up- regulated compared to the serum condition (Table S1) (See Supplementary Online Information at www.celljournal. org). Proteins up-regulated under 2i and R2i conditions are highly enriched for the terms associated with oxidation- reduction, amino acid and lipid metabolism, glycolysis, translation, mRNA processing and metabolic processes (Fig .2A). 

Cellular oxidation-reduction (redox) status is regulated by metabolic activities and impacts numerous BP. Redox, which mainly occurs during the respiratory chain, is crucial in stem cell fate regulation (14). In this study, proteins such as succinate dehydrogenase (Sdhb), which catalyzes the oxidation of succinate to fumarate; in addition to ubiquinol cytochrome c reductase core protein 2 (Uqcrc2), which catalyzes the reduction of cytochrome c by the oxidation of coenzyme Q; cytochrome c oxidase assembly protein 15 (Cox15); and superoxide dismutase 1 (Sod1) up- regulated under 2i and R2i conditions (Fig .2B), which controlled the generation and scavenging of reactive oxygen species (ROS). 

**Fig.3:**
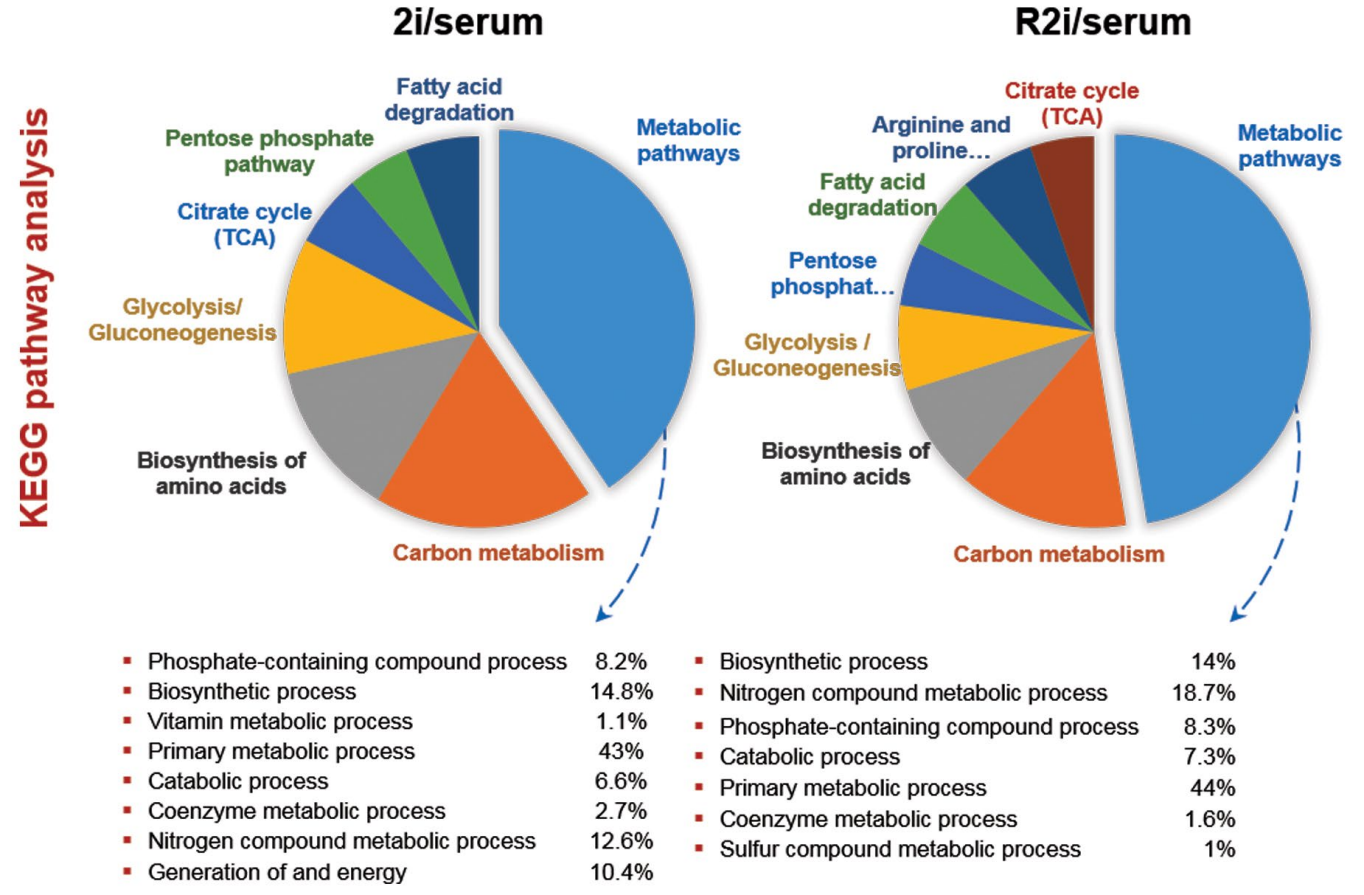
Kyoto Encyclopedia of Genes and Genomes (KEGG) pathway analysis of up-regulated proteins in 2i and R2i versus serum.

We observed more proteins associated with amino acid and lipid metabolism in 2i-and R2i-grown cells compared to serum. Asparagine synthetase (Asna), glutamic-oxaloacetic transaminase 1 (Got1), pyrroline-5-carboxylate reductase 1 (Pycr1), and serine hydroxymethyltransferase 2 (Shmt2), up-regulated under 2i and R2i conditions. Phosphoserine phosphatase (Psph) and argininosuccinate synthetase 1 (Ass1) were higher in 2i versus serum (Table S1) (See Supplementary Online Information at www.celljournal.org) and have been shown to be involved in serine, glycine, threonine and proline synthesis. ESCs have distinct epigenetic properties in terms of histone modifications in comparison with somatic cells. Metabolic flux analysis has indicated that threonine, by contribution in the synthesis of other amino acid provides fuel for ESC divisions and epigenetic regulation (15). KEGG pathway analysis showed that the abundance of proteins associated with metabolic process showed that phosphate-containing compounds, biosynthesis, vitamin metabolic, primary metabolic, generation of energy and coenzyme metabolic processes were higher in 2i and R2i conditions (Fig .3). 

### Generation of precursor metabolites and energy 

Several metabolic pathways are involved in energy production such as glycolysis and the tricarboxylic acid (TCA) cycle. Functional annotation of differentially expressed proteins by GO has shown accumulation of high levels of phosphofructokinase (Pfkp), phosphoenolpyruvate carboxykinase (Pck1), and phosphoglycerate kinase 1 (Pgk1) activities in 2i and R2i culture conditions, which indicated active glycolysis and glycogenesis. Fructose-bisphosphate aldolase A (Aldoa), another protein, increasesd under 2i and R2i conditions. This protein played a key role in glycolysis, as well as synthesis of D-glyceraldehyde 3-phosphate and glycerone phosphate from D-glucose (Table S1) (See Supplementary Online Information at www.celljournal.org). 

**Fig.4:**
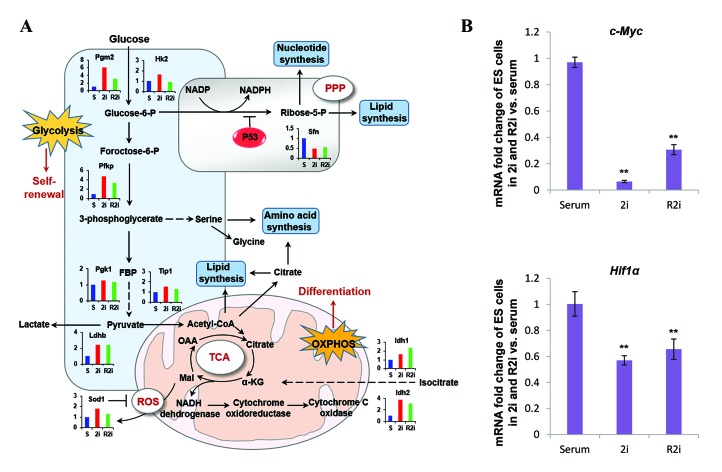
Metabolic pathways for stemness maintenance. A. By the glycolysis pathway, glucose metabolizes to pyruvate, which either undergoesreduction by lactate dehydrogenase (LDH) to lactate in the absence of oxygen or enters the mitochondria to be decarboxylated by pyruvatedehydrogenase to acetyl-CoA (AcCoA) in the presence of oxygen. In mitochondria, AcCoA form citrate by condensing oxaloacetate and can betransferred to the cytosol to provide carbon for lipid biosynthesis. The catalytic reactions of glycolysis provide several intermediates essentialfor the production of de novo nucleotides, phospholipids, and amino acids and B. Relative expression levels of c-Myc and Hif1a in 2i-, R2i, and serum-grown cells. (qRT-PCR, n=3, *; P<0.05, and **; P<0.01). Each mRNA expression level in the cells was normalized to the GAPDH housekeeping gene.

Hexokinase (HK) and lactate dehydrogenase (LDH) highly expressed in 2i and R2i-grown cells (Fig .4A). In the glycolysis pathway glucose metabolized to pyruvate, which either undergoes reduction by LDH to lactate or enters the mitochondria to be decarboxylated by pyruvate dehydrogenase to acetyl-CoA (AcCoA). The intermediates produced by glycolysis such as glucose-6-phosphate (G6P), fructose-6-phosphate (F6P), and glyceraldehyde-3-phosphate (G3P) are essential for the generation of nucleotides (via the pentose phosphatase pathway, PPP) (6). Other byproducts of glycolysis lactate contribute to anabolic and ATP-producing processes (16). PSCs prefer high rate of glycolysis for energy production rather than OXPHOS because proliferation requires significant amounts of nucleotides, amino acids, and lipids (2). Serum, as a naïve state condition, has also shown an up-regulation of some proteins involved in glycolysis. Some reprogramming factors, such as c-Myc and Hif1a and signaling network molecules that include AKT, PI3K, and mTOR promotes glycolysis and metabolic fluxes (17). Quantitative reverse transcription- polymerase chain reaction (qRT-PCR) analysis in the current study showed increased c-Myc and Hif1a expressions in serum compared to 2i and R2i culture conditions (Fig .4B). 

### Rapid cell cycling under 2i and R2i conditions

**Fig.5:**
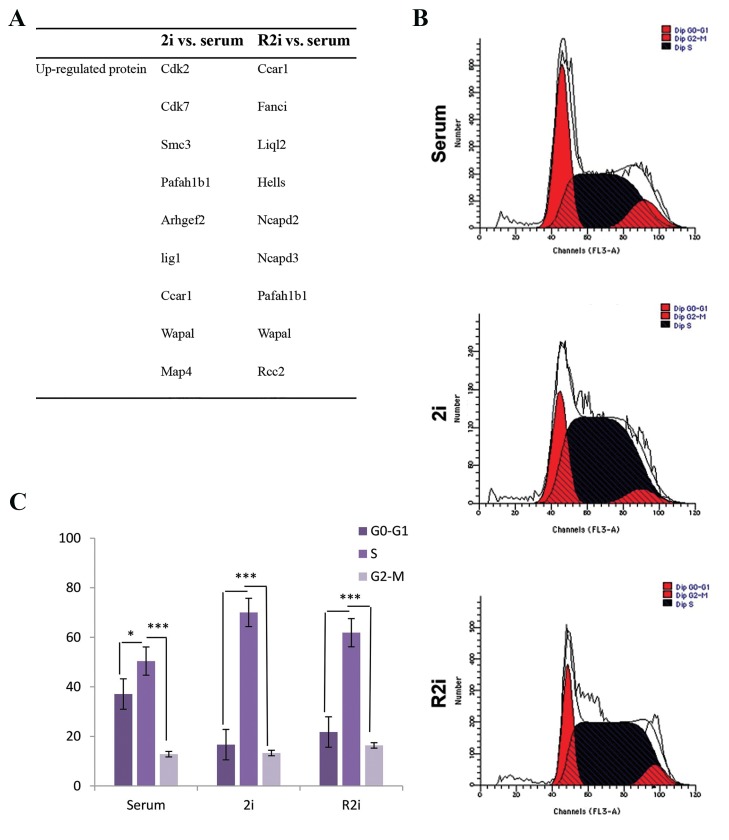
Increased related cell cycle proteins underground state pluripotency. A. Up-regulation of proteins involved in the cell cycle and cell division in 2i- and R2i-grown cells versus serum, B. Flow cytometry analysis of serum-, 2i-, and R2i-grown cells, and C. Quantified flow cytometry results showed the proportion of cells in the G1, S, and G2 phases of the cell cycle. Values are mean ± SD (n=3, *; P<0.05, and ***; P<0.01).

We observed changes in the expression levels of several proteins involved in the cell cycle and cell proliferation Cdk2, Cdk7, Fanci, Wapal, Anax11, Ccar1, Ligl2, Ncapd2, Ncapd3, Rcc2, Hells, and Pafah1b1 in 2i and R2i versus serum conditions (Fig .5, Fig Table S1) (See Supplementary Online Information at www.celljournal.org). Cdk2 and Cdk7 up-regulated under the 2i condition have been shown to play a specific role in the maintenance of pluripotency. We confirmed the shotgun proteomics data by flow cytometry analysis of cell cycle distribution. The results showed a significant accumulation of cells in S phase, 70% in 2i-grown cells and 61% in R2i-grown cells. The proportion of serum-grown cells increased in the G0/ G1 phase. The sub-G1 phase (apoptotic cells) did not show any changes between the conditions (Fig .5B, C). 

## Discussion

In this study, we have used the shotgun proteomics approach to show that 2i and R2i-grown cells exhibited protein up-regulation of oxidation-reduction, amino acid and lipid metabolism, glycolysis, translation, mRNA processing, cell cycle and metabolic processes compared to the serum condition. The oxidative status is regulated by the controlled balance of ROS production and scavenging through the reduction in oxidation substrate and up- regulation of antioxidant enzyme (18). Low levels of ROS mediate the proliferation of PSCs by the activation of several key cellular pathways such as extracellular signal-regulated kinases (ERK) 1/2 MAPK, nuclear factor-..B (NF-..B), and Wnt signaling (14). Recently it has been demonstrated that ROS modulates Oct4 posttranslational modifications, leading to the enhanced nuclear localization of Oct4 (19). ROS also mediates the lineage-specific differentiation of PSCs, so the balance between ROS generation and scavenging regulates the redox homeostasis in stem cells. Over expression of Sod1, as an antioxidant enzyme in 2i and R2i conditions, resultes in ROS scavenging and negative regulation of proliferation. 

2i and R2i-grown cells showed up-regulated proteins associated with amino acid and lipid metabolism. Amino acid metabolism in mESCs appears to modulate self- renewal, differentiation and the epigenetic process. Lipid metabolism plays an important role in cellular reprogramming (19). Other studies have reported that somatic cell reprogramming might be accompanied by a lipid metabolic shift from saturation to unsaturation (20, 21). Moussaieff et al. (22) observed that AcCoA, a key precursor of lipid synthesis, was important for maintaining histone acetylation in ESCs, which further extended the connection between metabolic intermediates and the regulation of open chromatin essential to the unique capacities of PSCs. 

We have shown that glycolysis was more prominent under 2i and R2i conditions. High glycolytic flux in PSCs allows for the quick generation of ATP and the pentose phosphate pathway (PPP) to generate ribose-5phosphate for nucleotides and NADPH-reducing power for nucleotide and lipid biosynthesis (23). Both ATP and nucleotides are required to power the rapid proliferation and DNA replication of ESCs (14). According to the current study results, under 2i and R2i culture conditions, the cells underwent rapid cell cycling with more than 60% of the population actively replicating DNA (S-phase). Only a small proportion remained in G1 and G2 phases. This agreed with an earlier study which reported that mESCs displayed rapid cell cycling along with a highly enriched proportion of S-phase and an unusually short G1 phase. Therefore, they particularly depended upon glycolysis to support cellular growth and division (10).

Expression levels of Cdk2 and Cdk7 proteins significantly up-regulated in 2i cells, which confirmed the cell cycle data. Cdk2 plays an important role in S phase progression by accociating with cyclin A, although CDK2 is a known effector of the G1 to S DNA damage checkpoint in mammalian cells (24). Cdk7 is essential for activation of the cell cycle through phosphorylation of key threonine residues in Cdk1 and Cdk2. CDKs can interact with epigenetic regulators involved in the maintenance of pluripotency, such as DNA methylase DNMT1 (25) and the higher-order chromatin organizer HP1a (26).

## Conclusion

This study revealed that mESCs cultured under 2i and R2i conditions used the glycolysis pathway for the generation of energy and intermediate products. 2i- and R2i-grown cells underwent rapid cell cycling as a feature of pluripotency by the overexpression of cell division and cell proliferation associated proteins. 

## Supplementary PDF


