# Supplementary material for: Metabolic Signature of Pluripotent Stem Cells
Source: Cell J. 2018 May 28;20(3):388–95. doi: 10.22074/cellj.2018.5514 (PMC6004998; doi:10.22074/cellj.2018.5514)
Supplement: Supplementary file 1 [file Cell-J-20-388-s01.pdf]

www.celljournal.org

Supplementary Information for

Metabolic Signature of Pluripotent Stem Cells

Sara Taleahmad, Ph.D.[1, 2], Seyedeh Nafiseh Hassani, Ph.D.[2], Ghasem Hosseini Salekdeh, Ph.D.[1, 3]*, Hossein Baharvand, Ph.D.[2, 4]*

1. Department of Molecular Systems Biology, Cell Science Research Center, Royan Institute for Stem Cell Biology and Technology, ACECR, Tehran, Iran
2. Department of Stem Cells and Developmental Biology, Cell Science Research Center, Royan Institute for Stem Cell Biology and Technology, ACECR, Tehran, Iran
3. Department of Systems Biology, Agricultural Biotechnology Research Institute of Iran, Karaj, Iran
4. Department of Developmental Biology, University of Science and Culture, ACECR, Tehran, Iran

**Corresponding Addresses: P.O.Box: 16635-148, Department of Molecular Systems Biology, Cell Science Research Center, Royan Institute for Stem Cell Biology and Technology, ACECR, Tehran, Iran*
P.O.Box: 16635-148, Department of Stem Cells and Developmental Biology, Cell Science Research Center, Royan Institute for Stem Cell Biology and Technology, ACECR, Tehran, Iran
Emails: salekdeh@royanInstitute.org, baharvand@royanInstitute.org

Table S1: List of up- regulated proteins between R2i and 2i versus serum

R2i versus serum					2i versus serum				
Pgm1	0.000135	5.74E-05	0.05	2.35	Lig1	0.0003214	0.0002097	0.05	1.53
Glb1	0.0001217	5.95E-05	0.05	2.05	Mrps17	0.0003511	7.76E-05	0.05	4.52
Renbp	0.0001076	3.03E-05	0.05	3.54	Ckb	0.0009502	0.0005902	0.05	1.61
Cox15	7.66E-05	2.26E-05	0.05	3.39	Plcb3	3.01E-05	1.05E-05	0.05	2.86
Pold2	7.58E-05	2.03E-05	0.05	3.73	Sbno1	2.46E-05	6.71E-06	0.05	3.66
Nipsnap1	0.0002916	0.0001804	0.05	1.62	Etfb	0.000539	0.0001136	0.05	4.75
Rbm7	0.0001304	4.84E-05	0.05	2.69	Ppp1r7	0.0002211	9.57E-05	0.05	2.31
Asah1	0.0001442	4.19E-05	0.05	3.44	Dnmt1	4.18E-05	2.16E-05	0.05	1.94
Amt	0.0003458	0.0001363	0.05	2.54	Hsp90b1	0.0028659	0.0019932	0.05	1.44
Smarcc1	0.0002581	0.0001366	0.05	1.89	Tigar	0.0006109	0.0003772	0.05	1.62
Clic4	0.000626	0.0005116	0.05	1.22	Cox15	0.0001236	2.27E-05	0.05	5.45
Akr7a5	0.0001067	2.53E-05	0.05	4.21	Cdk7	0.0001806	0.0001125	0.05	1.61
Pgd	0.0005854	0.0004638	0.05	1.26	Ndufs1	0.000213	8.61E-05	0.05	2.47
Fermt2	0.00013	9.83E-05	0.05	1.32	Canx	0.0013489	0.0009372	0.05	1.44
Sf3b1	0.0003709	0.0002744	0.04	1.35	Psmd5	0.0002211	0.0001232	0.04	1.80
Elavl1	0.0009206	0.0005227	0.04	1.76	Esyt1	7.08E-05	2.59E-05	0.04	2.74
Hdgf	0.0007758	0.0005599	0.04	1.39	Slc25a20	0.0003562	6.93E-05	0.04	5.14
Coro1b	9.58E-05	2.67E-05	0.04	3.60	Pgk1	0.0025007	0.0019652	0.04	1.27
Hnrnph3	0.0002302	9.54E-05	0.04	2.41	Prdx2	0.0027128	0.0020692	0.04	1.31
Cyb5r3	0.0008781	0.0004906	0.04	1.79	Cdk2	0.0002528	0.000155	0.04	1.63
Lars	0.0003676	0.0002092	0.04	1.76	Wapl	4.07E-05	2.02E-05	0.04	2.02
Timm44	0.0001332	3.67E-05	0.04	3.62	Cdv3	0.0004032	9.39E-05	0.04	4.29
Kras	0.0004108	8.58E-05	0.04	4.79	Isyna1	0.0003254	0.0001885	0.04	1.73
Slc25a5	0.0039652	0.0031383	0.04	1.26	Skiv2l2	0.0002199	6.51E-05	0.04	3.38
Iars	0.000347	0.0002051	0.04	1.69	Mcm6	0.0007446	0.0005096	0.04	1.46
Esyt1	7.94E-05	2.57E-05	0.04	3.08	Snx5	0.0001457	3.13E-05	0.04	4.66
Impa2	0.0006045	0.0002026	0.04	2.98	Asna1	0.0004725	0.0002515	0.04	1.88
Gars	0.0009249	0.0006513	0.04	1.42	Sod1	0.0027449	0.0015581	0.04	1.76
Mthfd2	0.0003764	0.0001917	0.04	1.96	Sdhb	0.0003203	9.79E-05	0.04	3.27
Skiv2l2	0.0001555	6.49E-05	0.04	2.40	Smc3	8.60E-05	2.81E-05	0.04	3.06
Ilf2	0.0004379	0.0002159	0.04	2.03	Hspa9	0.0019751	0.0013765	0.04	1.43
Hars	0.0002269	0.0001045	0.04	2.17	Sars2	6.30E-05	1.77E-05	0.04	3.56
U2surp	4.57E-05	2.74E-05	0.04	1.66	Prkcsh	0.0002373	0.0001381	0.04	1.72
Acat2	0.0002657	0.0001249	0.03	2.13	Calu	0.0001191	2.81E-05	0.04	4.24

Table S1: Continued

	R2i versus serum					2i versus serum			
Rab14	0.0010775	0.0008058	0.03	1.34	Pafah1b1	0.0001194	6.69E-05	0.04	1.78
Caml	0.0001322	5.74E-05	0.03	2.31	Kras	0.0004484	8.60E-05	0.03	5.21
Pfas	0.0004521	0.0002708	0.03	1.67	Ldhb	0.0014666	0.0006105	0.03	2.40
Hadh	0.0017938	0.0008967	0.03	2.00	Usp10	0.0001022	2.63E-05	0.03	3.89
Slc12a7	5.02E-05	1.21E-05	0.03	4.14	Slc1a5	7.01E-05	1.79E-05	0.03	3.91
Rab10	0.0010358	0.0008376	0.03	1.24	Ldah	0.0001376	6.15E-05	0.03	2.24
Smarca4	4.24E-05	2.40E-05	0.03	1.77	Cul5	3.72E-05	1.95E-05	0.03	1.90
Mrpl15	0.0001772	6.78E-05	0.03	2.61	Asah1	0.0001669	4.21E-05	0.03	3.96
Srrm2	2.11E-05	5.07E-06	0.03	4.16	Nelfb	5.43E-05	1.58E-05	0.03	3.43
Aars	0.0010562	0.0007546	0.03	1.40	Actr10	8.62E-05	2.23E-05	0.03	3.86
Aco2	0.0002884	0.0001772	0.03	1.63	Pabpn1	0.0001524	3.22E-05	0.03	4.73
Sgpl1	4.87E-05	1.64E-05	0.03	2.97	Copz1	0.0005185	0.0002784	0.03	1.86
Slc25a20	0.0005093	6.90E-05	0.03	7.39	ND4	0.0004092	0.0001887	0.03	2.17
Anxa11	0.0001338	8.05E-05	0.03	1.66	Srsf6	0.0002689	0.0001233	0.03	2.18
Gga2	5.99E-05	1.58E-05	0.03	3.79	Psph	0.0004298	0.0001409	0.03	3.05
Ndufv2	0.000144	3.82E-05	0.03	3.77	Myl12b	0.0003817	7.35E-05	0.03	5.19
Ncapd3	1.83E-05	6.11E-06	0.03	3.00	Nsfl1c	0.0002406	8.64E-05	0.03	2.78
Tsfm	0.0003092	0.0001351	0.03	2.29	Vat1	0.0001773	6.26E-05	0.03	2.83
Pleckstrin	2.96E-05	8.79E-06	0.03	3.37	Rps9	0.0032942	0.0029339	0.03	1.12
Galk1	0.0007194	0.0003862	0.03	1.86	Pgd	0.000607	0.0004658	0.03	1.30
Dcps	0.0002479	0.0001223	0.03	2.03	Pdia4	0.0006976	0.0003954	0.03	1.76
Acin1	4.82E-05	9.61E-06	0.03	5.01	Prmt1	0.0009174	0.0007494	0.02	1.22
Mvd	8.94E-05	2.34E-05	0.02	3.82	Parp1	0.0001866	6.45E-05	0.02	2.89
Aifm1	0.0001836	7.17E-05	0.02	2.56	Tmx2	0.0003558	0.0001775	0.02	2.01
Calu	9.50E-05	2.80E-05	0.02	3.40	Plaa	0.0002079	0.0001311	0.02	1.59
Rab5b	0.000515	0.0003056	0.02	1.69	Aldoa	0.0044785	0.0031054	0.02	1.44
Rcc2	0.0006915	0.0005298	0.02	1.31	Amt	0.000334	0.0001369	0.02	2.44
Hprt	0.0012327	0.0009354	0.02	1.32	Ero1l	0.0001512	2.66E-05	0.02	5.68
Myo1b	3.94E-05	7.79E-06	0.02	5.06	Aifm1	0.000195	7.21E-05	0.02	2.71
Pafah1b1	0.0001103	6.67E-05	0.02	1.65	Pitrm1	0.0001089	4.43E-05	0.02	2.46
Scpep1	0.0001016	4.46E-05	0.02	2.28	Eif4b	0.000445	0.0002622	0.02	1.70
Fdps	0.0008785	0.0006277	0.02	1.40	Psmd3	0.0007135	0.0004341	0.02	1.64
Ero1l	0.0001634	2.66E-05	0.02	6.15	Naxe	0.0001997	7.38E-05	0.02	2.71
Parp1	0.0001656	6.43E-05	0.02	2.58	Xpo4	7.59E-05	1.11E-05	0.02	6.81

Table S1: Continued

R2i versus serum					2i versus serum				
Nampt	0.0001375	8.61E-05	0.02	1.60	Bsg	0.0004835	6.28E-05	0.02	7.70
Psmc4	0.0009013	0.0005993	0.02	1.50	Ogdhl	0.0001367	1.59E-05	0.02	8.61
Llgl2	4.91E-05	9.05E-06	0.02	5.43	Atp2a2	0.0003749	0.0001755	0.02	2.14
Ldhb	0.0014798	0.0006077	0.02	2.44	Pdia6	0.0009001	0.0005514	0.02	1.63
Atp5a1	0.0016994	0.0013572	0.02	1.25	Pgm1	0.0001923	5.76E-05	0.02	3.34
Wapl	6.26E-05	2.01E-05	0.02	3.12	Yars	0.000333	0.0001555	0.02	2.14
Ckb	0.0012344	0.0005875	0.02	2.10	Coro1b	0.0001675	2.67E-05	0.02	6.27
Cstf3	0.0001271	4.23E-05	0.02	3.01	Mrps9	0.0002028	7.86E-05	0.02	2.58
Otub1	0.0003874	0.0001515	0.02	2.56	Adh5	0.0006507	0.0003416	0.02	1.90
Acp6	0.0001338	3.06E-05	0.02	4.37	Arhgef2	4.64E-05	2.11E-05	0.02	2.20
Aldoa	0.003942	0.0030923	0.02	1.27	Rbm26	4.81E-05	5.63E-06	0.02	8.55
Naxe	0.0001658	7.35E-05	0.02	2.26	Xrn2	4.24E-05	2.87E-05	0.02	1.48
Akap1	3.41E-05	6.75E-06	0.02	5.05	Echs1	0.0003964	0.0001651	0.02	2.40
Ccar1	2.99E-05	7.79E-06	0.02	3.83	Tomm70a	0.0002081	0.000114	0.02	1.83
Gcdh	8.75E-05	2.09E-05	0.02	4.18	Slc2a3	0.0010215	0.000604	0.02	1.69
Eif5b	0.0001637	9.81E-05	0.02	1.67	Cpt1a	4.64E-05	1.18E-05	0.01	3.93
Prkag1	0.0001597	6.07E-05	0.02	2.63	Rpl35	0.0025232	0.0014195	0.01	1.78
Rpf2	0.0001378	7.23E-05	0.02	1.91	Akap12	0.0003917	0.0002101	0.01	1.86
Slc25a13	0.0005985	0.0002756	0.02	2.17	Rfc2	0.0001598	8.06E-05	0.01	1.98
Fkbp5	0.0001183	4.49E-05	0.02	2.64	Nedd4	0.0005116	0.0003099	0.01	1.65
Taldo1	0.001254	0.0007148	0.02	1.75	Lrpprc	0.0003741	0.0001716	0.01	2.18
Etfa	0.0011664	0.0003961	0.02	2.94	Pgm2	0.0004393	7.14E-05	0.01	6.15
Sart3	0.0001073	3.22E-05	0.01	3.34	Calr	0.0016756	0.0011118	0.01	1.51
Rpap1	3.60E-05	6.72E-06	0.01	5.36	Phb	0.0026436	0.0018071	0.01	1.46
RNA-	0.0002869	3.70E-05	0.01	7.76	Gm10705	0.0015344	0.0012888	0.01	1.19
Rfc5	0.0002014	0.0001031	0.01	1.95	Nup85	0.0001194	6.39E-05	0.01	1.87
Nup214	3.41E-05	1.27E-05	0.01	2.69	Wdr18	0.000251	0.0001717	0.01	1.46
Psmd5	0.0002915	0.0001227	0.01	2.38	Diaph1	0.0001249	6.13E-05	0.01	2.04
Sf3b2	7.40E-05	1.48E-05	0.01	4.99	Myo1b	4.21E-05	7.82E-06	0.01	5.38
Fxr2	9.21E-05	1.95E-05	0.01	4.71	Pgpep1	0.0002887	0.0001002	0.01	2.88
Dnajc7	0.0002309	9.91E-05	0.01	2.33	Pfas	0.0004608	0.000272	0.01	1.69
Nelfb	6.62E-05	1.58E-05	0.01	4.19	Pgam1	0.0049751	0.003787	0.01	1.31
Crk	0.0001402	6.72E-05	0.01	2.09	Set	0.0020568	0.0014015	0.01	1.47
Actl6a	0.0002742	0.0001532	0.01	1.79	Aldh2	0.0004545	0.0002837	0.01	1.60

Table S1: Continued

R2i versus serum					2i versus serum				
Snrpa1	0.0007785	0.0004469	0.01	1.74	Scpep1	0.0001009	4.48E-05	0.01	2.25
Aldh2	0.0003978	0.0002825	0.01	1.41	Gcdh	0.0001249	2.10E-05	0.01	5.95
Uggt1	0.0002006	0.0001352	0.01	1.48	Lbr	0.0001792	0.0001135	0.01	1.58
Pck2	0.0001316	1.40E-05	0.01	9.39	Fkbp5	0.0001626	4.51E-05	0.01	3.61
Sf3a1	0.0001848	8.18E-05	0.01	2.26	Hnrnpll	0.0001504	6.81E-05	0.01	2.21
Pabpn1	0.0001733	3.20E-05	0.01	5.41	Eci1	0.0005045	0.0002646	0.01	1.91
Lrpprc	0.0002819	0.0001709	0.01	1.65	Ass1	0.0006549	0.0003071	0.01	2.13
Pold1	0.0002402	4.18E-05	0.01	5.74	Slc25a13	0.0007079	0.0002767	0.01	2.56
Hells	4.07E-05	6.53E-06	0.01	6.23	Asns	0.0006564	0.0003593	0.01	1.83
P4ha1	5.83E-05	1.02E-05	0.01	5.72	Hspa4	0.0018103	0.0014335	0.01	1.26
Qprt	0.0001122	1.97E-05	0.01	5.69	Msh6	0.0006953	0.0003794	0.01	1.83
Nop58	0.0001261	5.15E-05	0.01	2.45	Aldoart1	0.0016993	0.0011323	0.01	1.50
Msh6	0.0006565	0.0003777	0.01	1.74	Suclg2	0.0001658	2.22E-05	0.01	7.46
Usp9x	0.0001795	0.0001049	0.01	1.71	Aco2	0.0003829	0.000178	0.01	2.15
Shmt2	0.0005413	0.0002207	0.01	2.45	Pycr1	0.0003143	3.19E-05	0.01	9.87
Got1	0.0012227	0.000569	0.01	2.15	Shmt1	0.0002651	0.0001623	0.01	1.63
Sars	0.0007798	0.0003583	0.01	2.18	Cfdp1	0.0002833	3.16E-05	0.01	8.96
Diaph1	0.0001955	6.10E-05	0.01	3.20	Lonp1	0.0002308	3.73E-05	0.01	6.19
Pycr1	0.0001854	3.18E-05	0.01	5.84	Smarca4	3.64E-05	2.41E-05	0.01	1.51
Copa	0.0002041	0.0001273	0.01	1.60	Tsfm	0.0002823	0.0001357	0.01	2.08
Ncapd2	0.0002647	0.0002091	0.01	1.27	Dcps	0.0002555	0.0001229	0.01	2.08
Upp1	0.0013234	0.0002969	0.01	4.46	Nipsnap2	0.0001272	1.90E-05	0.01	6.71
Suclg2	0.0001463	2.21E-05	0.01	6.62	Aars	0.0014658	0.0007579	0.01	1.93
Adh5	0.0007601	0.0003402	0.01	2.23	Pmvk	0.0004548	3.18E-05	0.00	14.29
Eif4g2	3.56E-05	6.35E-06	0.01	5.60	Dnpep	0.0002189	0.0001074	0.00	2.04
Rab5a	0.0007946	0.0004652	0.01	1.71	Cars	0.0002711	9.44E-05	0.00	2.87
Metap2	6.56E-05	1.17E-05	0.01	5.59	Got1	0.0013309	0.0005714	0.00	2.33
As3mt	8.48E-05	1.49E-05	0.01	5.70	Mrpl15	0.0002065	6.81E-05	0.00	3.03
Acat1	7.91E-05	1.39E-05	0.01	5.70	Supt16	0.0001005	3.65E-05	0.00	2.76
Ccdc12	0.000293	9.86E-05	0.01	2.97	Uqcrc2	0.0007223	0.0003244	0.00	2.23
Pfkp	0.0003035	9.00E-05	0.00	3.37	Aldh18a1	0.0003277	0.0001127	0.00	2.91
Rab1a	0.0028683	0.0023457	0.00	1.22	Hk2	0.0007059	0.0004283	0.00	1.65
Idh1	0.0005199	0.0002176	0.00	2.39	Slc3a2	0.0008617	0.0005178	0.00	1.66
Cars	0.0002507	9.40E-05	0.00	2.67	Map2k1	0.0002655	8.16E-05	0.00	3.25

Table S1: Continued

R2i versus serum					2i versus serum				
Atp5c1	0.0007108	0.0004595	0.00	1.55	Isoc2a	0.0012653	0.0002878	0.00	4.40
Dhrs7	8.21E-05	1.63E-05	0.00	5.03	Nop58	0.0001183	5.17E-05	0.00	2.29
Mdh2	0.0022925	0.0014363	0.00	1.60	Mdh2	0.0018758	0.0014423	0.00	1.30
Alpl	0.0001397	6.14E-05	0.00	2.28	Fip1l1	6.63E-05	1.04E-05	0.00	6.39
Mgea5	5.04E-05	1.81E-05	0.00	2.79	Pck2	0.0001901	1.41E-05	0.00	13.52
Cfdp1	0.000258	3.15E-05	0.00	8.20	Alpl	0.0001913	6.16E-05	0.00	3.10
Khsrp	0.0004069	0.0002618	0.00	1.55	Pfkp	0.0004272	9.04E-05	0.00	4.73
Morc3	2.92E-05	5.83E-06	0.00	5.01	Fn1	1.64E-05	2.50E-06	0.00	6.54
Supt16	0.0001233	3.63E-05	0.00	3.40	Rfc5	0.0002229	0.0001035	0.00	2.15
Ints4	2.86E-05	5.75E-06	0.00	4.97	Map4	6.44E-05	5.31E-06	0.00	12.12
Dhx57	2.06E-05	4.15E-06	0.00	4.96	Trap1	0.0011306	0.0008163	0.00	1.39
Nt5c3b	0.0001035	2.08E-05	0.00	4.96	Acaa1a	0.0003489	0.0001186	0.00	2.94
Tbcb	0.0002942	0.0001288	0.00	2.28	Prkag1	0.0002935	6.10E-05	0.00	4.81
Rps10	0.0031389	0.0025317	0.00	1.24	Ccdc12	0.0001941	9.90E-05	0.00	1.96
Asns	0.0005315	0.0003579	0.00	1.49	Idh2	0.000661	0.000176	0.00	3.76
Usp48	8.14E-05	5.15E-06	0.00	15.80	P4ha1	0.0001	1.02E-05	0.00	9.76
Idh2	0.0005491	0.0001752	0.00	3.13	Mthfd1l	0.0004711	0.0002774	0.00	1.70
Lap3	0.0005773	0.0003284	0.00	1.76	Ferritin	0.0001902	2.52E-05	0.00	7.56
Ivd	8.49E-05	1.34E-05	0.00	6.33	Sars	0.0009207	0.0003598	0.00	2.56
Map2k1	0.0002906	8.13E-05	0.00	3.58	Tpi1	0.0026046	0.0017118	0.00	1.52
Myo18a	2.07E-05	2.69E-06	0.00	7.70	Eif4g2	4.61E-05	6.37E-06	0.00	7.23
Isoc2a	0.0009665	0.0002866	0.00	3.37	Sec61a2	0.0003092	1.20E-05	0.00	25.87
Sec61a2	0.0002017	1.19E-05	0.00	16.94	Shmt2	0.0005526	0.0002216	0.00	2.49
Fanci	8.43E-05	4.02E-05	0.00	2.10	Dhrs7	9.63E-05	1.64E-05	0.00	5.88
Kif15	3.24E-05	1.16E-05	0.00	2.78	Nup35	0.0001109	1.88E-05	0.00	5.88
Plin3	0.0004773	0.0002059	0.00	2.32	Tuba1c	0.0059271	1.25E-05	0.00	474.03
Acaa2	0.0002036	1.49E-05	0.00	13.70					
Pola1	3.09E-05	1.11E-05	0.00	2.77					
Amdhd2	8.08E-05	1.43E-05	0.00	5.66					
Cndp2	0.0003328	0.0001453	0.00	2.29					
Ppm1b	7.53E-05	1.19E-05	0.00	6.32					
Mfge8	6.83E-05	1.21E-05	0.00	5.63					
Zmym2	2.26E-05	4.02E-06	0.00	5.63					
Gtf2b	0.0001006	1.79E-05	0.00	5.63					
Rbm26	3.16E-05	5.60E-06	0.00	5.63					
Gna13	8.90E-05	1.41E-05	0.00	6.30					
HORMA	0.0001839	7.90E-05	0.00	2.33					
Krt76	4.94E-05	9.89E-06	0.00	4.99					
Mindy3	6.25E-05	1.25E-05	0.00	4.99					
Polr3c	5.11E-05	1.02E-05	0.00	4.99					
Tuba3b	0.0044708	1.25E-05	0.00	359.07					
Tuba1c	0.0068569	1.25E-05	0.00	550.70					
Aldoc	0.0004767	1.58E-05	0.00	30.23					
